# Two-Photon Holographic Stimulation of ReaChR

**DOI:** 10.3389/fncel.2016.00234

**Published:** 2016-10-18

**Authors:** Emmanuelle Chaigneau, Emiliano Ronzitti, Marta A. Gajowa, Gilberto J. Soler-Llavina, Dimitrii Tanese, Anthony Y. B. Brureau, Eirini Papagiakoumou, Hongkui Zeng, Valentina Emiliani

**Affiliations:** ^1^Wavefront-Engineering Microscopy Group, Neurophotonics Laboratory, Centre National de la Recherche Scientifique UMR8250, Paris Descartes UniversityParis, France; ^2^Allen Institute for Brain ScienceSeattle, WA, USA; ^3^Institut National de la Santé et de la Recherche Médicale (INSERM)Paris, France

**Keywords:** optogenetics, 2-photon excitation, computer generated holography, opsin, action-potential generation, neuroscience, cortex

## Abstract

Optogenetics provides a unique approach to remotely manipulate brain activity with light. Reaching the degree of spatiotemporal control necessary to dissect the role of individual cells in neuronal networks, some of which reside deep in the brain, requires joint progress in opsin engineering and light sculpting methods. Here we investigate for the first time two-photon stimulation of the red-shifted opsin ReaChR. We use two-photon (2P) holographic illumination to control the activation of individually chosen neurons expressing ReaChR in acute brain slices. We demonstrated reliable action potential generation in ReaChR-expressing neurons and studied holographic 2P-evoked spiking performances depending on illumination power and pulse width using an amplified laser and a standard femtosecond Ti:Sapphire oscillator laser. These findings provide detailed knowledge of ReaChR's behavior under 2P illumination paving the way for achieving in depth remote control of multiple cells with high spatiotemporal resolution deep within scattering tissue.

## Introduction

Optogenetics has revolutionized neuroscience by enabling remote activation or inhibition of specific populations of neurons in intact brain preparations through genetically-targeted, light-sensitive channels and pumps (Nagel et al., 2003; Boyden et al., 2005; Adamantidis et al., 2015). Despite great advances in opsin engineering and photoactivation methods achieved over the last decade, studying the role of individual neurons within neuronal circuits is still a challenge as it requires photo-stimulation of one or several individually chosen cells within scattering tissues (Peron and Svoboda, 2011; Emiliani et al., 2015), with millisecond time precision and micrometer spatial resolution. Recent developments of *ad hoc* opsins (Mattis et al., 2012; Klapoetke et al., 2014) and innovative illumination approaches (reviewed in Papagiakoumou, 2013; Bovetti and Fellin, 2015) offer the possibility of tackling these challenges.

Red-shifted variants of channelrhodopsin, such as C1V1 (Yizhar et al., 2011), ReaChR (Lin et al., 2013; Hooks et al., 2015) or Chrimson (Klapoetke et al., 2014), with spectral peaks near and above 600 nm, enable deeper brain stimulation relative to blue-green shifted opsins. For instance, red-orange light illumination of ReaChR, has permitted *in vivo* trans cranial optogenetics in deep brain structures (Lin et al., 2013). However, in depth neuronal stimulation using visible light does not enable cellular resolution.

In depth optogenetics at the single cell level requires the use of two-photon (2P) stimulation. However, the small conductance [~40 fS for ChR2 (Feldbauer et al., 2009)], of most existing optogenetic actuators and the limited number of channels contained in the micro-sized illumination volume renders 2P-optogenetics challenging. This has prompted the design of new 2P-illumination approaches for optimized photocurrent integration, each with its advantages and limitations. Two-photon scanning approaches optimize current integration by quickly scanning a micrometer-diameter spot across the cell body faster than the channel's closing time (Rickgauer and Tank, 2009; Andrasfalvy et al., 2010; Packer et al., 2012; Prakash et al., 2012). On the contrary, 2P-parallel approaches enable synchronous current integration from all illuminated channels by delivering light simultaneously on axially confined, user-defined regions (Papagiakoumou et al., 2010, 2013; Bègue et al., 2013). Intermediate solutions, using scanning of a low numerical aperture beam (Rickgauer et al., 2014) or multiplexed holographic beams (Packer et al., 2015), have also been developed.

Either approach, combined with red-shifted opsins could enable optimal deep-brain photo-stimulation with cellular resolution. Parallel approaches illuminate all targets simultaneously and provide a higher temporal resolution than scanning methods: action potential (AP) generation is achieved with millisecond temporal resolution (1–10 ms) with a parallel approach (Bègue et al., 2013) whereas it requires 5–70 ms with scanning (Rickgauer and Tank, 2009; Andrasfalvy et al., 2010; Packer et al., 2012; Prakash et al., 2012). However, parallel approaches divide the available laser power among all targets. As a consequence, the maximum area photo-excited within a single illumination pattern, and therefore the maximum number of target cells, is limited by the available laser power and the cross-section of the opsin. Consequently simultaneous, multiple cell targeting requires using high cross-section opsins and efficient illumination methods.

To date, 2P activation of red-shifted opsins has only been demonstrated for C1V1 (Packer et al., 2012, 2015; Prakash et al., 2012; Bègue et al., 2013; Rickgauer et al., 2014). ReaChR offers improved membrane trafficking, as well as higher photocurrent for 1P stimulation (Lin et al., 2013). Therefore, ReaChR has the potential to be highly sensitive for 2P stimulation.

Here, we characterized the 2P absorption spectrum and kinetics parameters of ReaChR in cultured cells and in acute brain slices. We demonstrated that using computer generated holography with an amplified laser combined with ReaChR enables 2P generation of photo-currents in the range of one nanoampere. Moreover, we demonstrated reliable action potential generation with millisecond temporal resolution and sub-millisecond temporal precision (jitter). These results open new possibilities for in depth, simultaneous 2P stimulation of multiple targets.

## Materials and methods

### ReaChr expression in biological samples

#### Cultured cells

ReaChR was first expressed in cells in culture. We used Chinese Hamster Ovary (CHO) cells as they have few gap junctions which would interfere with measurements of currents for single cell photo-stimulation, lowering their amplitude and slowing kinetics (Conti et al., 2016). CHO cells were cultured in an incubator at 37°C and 5% CO_2_ in a D-MEM/F12 GlutaMAX medium (Life Technologies) with the addition of 1 mM glutamine, 1% streptomycin and 10% fetal bovine serum. Cells were plated on Thermanox plastic coverslips (Thermo Scientific) 24 h prior to transfection. The DNA was transfected using the EX-Gen 500 transfection reagent and cells were recorded 24–48 h after transfection. The plasmid used had a p2A sequence (Prakash et al., 2012) which allowed for the simultaneous expression of the photochannel and the YFP marker but independent targeting (pAAV-ReaChR-p2A-eYFP) (provided by the Allen Institute). CHO cells were transfected with pAAV-ReaChR-p2A-EYFP, and examined 2–3 days after transfection.

#### Brain slices

##### Virus injections

ReaChR was expressed in mouse brain tissue using AAVs as vectors. All experimental procedures were approved by the Paris Descartes Ethics Committee for Animal Research (registered number CEEA34.EV.118.12) and institutional guidelines of the care and use of laboratory animals (Council directive 86/609 EEC). Swiss male mice (Janvier, France) were injected 25–35 days post-natal with AAV1 CamKII-ReaChR-p2A-YFP or AAV1 Ef1α-ReaChR-p2A-dTomato (*n* = 94 mice). The dimer of Tomato (dTomato) (Shaner et al., 2004) was used in the latter construct to meet AAV genome size constraints. Mice were anesthetized with a mixture of ketamine (80 mg/kg body weight; Ketamine 1000, Virbac France) and xylazine (10 mg/kg body weight; Xylazine Rompun 2%, Bayer Healthcare) via intraperitoneal injection. They were placed in a stereotactic frame and the head was stabilized with ear bars and a mouth holder. Eyes were covered with lubricant (Dexpanthenol; Chauvin Ankerpharm GmbH Berlin) to prevent dehydration. The scalp was locally anesthetized with 0.03 mL lidocaine (Xylovet 21.33 mg/mL; CEVA). The skull was exposed by an incision of ~0.5 cm length on the anterior-posterior axis of the scalp above the visual cortex. The injection site was located 3.5 mm posterior of bregma and 2 mm lateral. A craniotomy of ~1 mm^2^ was performed and the dura mater was cut through. A 36-gauge stainless steel beveled needle (Coopers Needleworks Ltd) was placed at a depth of 200 μm from the brain surface and a volume of 1.5 μL AAV (total genome copies: 1.5 10^12^ to 1.5 10^14^) was injected at a rate of 100 nL per minute. After injection, the scalp was closed with cyanoacrylate (super glue Loctite; Henkel). Finally, a peritoneal injection of Antisedan (0.2 mg/kg; Janssen) and a subcutaneous injection with 0.2 mL of 9% saline solution were performed to allow for quick recovery from anesthesia.

To check for expression, 2 mice were fixed with PFA. They were deeply anesthetized with Pentobarbital 0.2 mg/g mouse. After clearing the blood with PBS though transcardiac perfusion, 4% PFA was used to fixate the animal. The fixed brain was removed, post-fixed in PFA for 1 h, and then washed with PBS. The fixed brains were sliced into 50 μm thick sagittal sections and mounted on glass cover slips for confocal imaging.

##### Acute brain slice preparation

Three hundred micrometer thick parasagittal slices, were prepared 4–10 weeks after viral injection, in accordance with European guidelines. Mice were decapitated. Slices were prepared using a Leica VT1200S slicer. The sucrose slicing solution contained (in millimoles of compound used to make a liter of solution): 25 glucose, 85 NaCl, 65 sucrose, 0.5 CaCl_2_, 4 MgCl_2_, 2.5 KCl, 1.25 NaH_2_PO_4_ and 26 NaHCO_3_. It was saturated with 95% O_2_/5% CO_2_. Slices were then incubated at 32–33°C for 30 min in a sucrose recovery solution containing (in millimoles of compound used to make a liter of solution): 25 glucose, 115 sucrose, 1 CaCl_2_, 2.5 MgCl_2_, 105 NaCl, 2.5 KCl, 1.25 NaH_2_PO_4_ and 26 NaHCO_3_ saturated with 95% O_2_/5% CO_2_.

### Imaging

#### Widefield IR and fluorescence system

For screening cells morphology and checking cell localization in acute brain slices we used a widefield infrared illumination system. This consisted of an IR-LED source (M780L2, Thorlabs) installed at the rear port of a SliceScope Scientifica microscope, an orientable blocking element to create oblique illumination and a condenser focusing the light on the sample. IR light transmitted through the sample was collected with an IR antireflection coated water-immersion objective (Nikon NIR MRD07420 N40X/0.80W) and sent to an IR CCD (IR-1000, DAGE-MIT).

For a first control of ReaChR expression, we performed widefield fluorescence imaging with a system comprising 2 interchangeable LED sources (Thorlabs M470L2, for YFP and M565L3 for dTomato) filtered by 2 interchangeable bandwidth excitation filters (Semrock FF01-452/45 for YFP and F01-545/55-25 for dTomato) and coupled to a diffuser (DG10-1500, Thorlabs) and an achromatic lens (*f* = 30 mm, #LA1805 Thorlabs). Fluorescence was collected through a tube lens (*f* = 200 mm), separated from excitation light using a dichroic mirror (Semrock FF510-Di02 for YFP and FF580-FDi01 for dTomato) and detected by a CCD camera (Orca-05G, Hamamatsu) after passing through a visible bandwidth filter (Semrock FF01-609/181 for YFP and FF01-665/150-25 for dTomato).

#### Two-photon scanning imaging system

For high-resolution fluorescence imaging we used a 2P raster scanning microscope consisting of a femtosecond tunable Laser (Coherent Chameleon Vision II, pulse width 140 fs, tuning range 680–1080 nm), relayed on a custom-made galvanometer-based scanhead (3 mm aperture, 6215H series, Cambridge Technology), imaged at the back aperture of an IR antireflection coated water-immersion objective (Nikon NIR MRD07420 N40X/0.80W) through an afocal telescope (scan lens: *f* = 100 mm, Thorlabs #AC508-100-B; tube lens: *f* = 300 mm, Thorlabs #AC508-300-B). For imaging of dTomato the Chameleon laser was tuned at 930 nm. Galvanometric mirrors were driven by two servo drivers (MicroMax series 671, Cambridge Technology) controlled by a Digital/Analog converter board (PCI-6110, National Instrument).

Epi-fluorescence signals were collected during imaging. Fluorescence photons were separated from excitation photons using a dichroic mirror (Semrock FF705-Di0) and a band-pass filter (Semrock FF01-750/SP) and then separated according to their wavelength using a second dichroic mirror (Semrock FF555-Di03). Red fluorescence was further isolated using a band-pass emission filter (Semrock FF02-617/73) and detected using a multi-alkali photomultiplier (Hamamatsu R9110). Green fluorescence light was further isolated using a blocking edge short-pass emission filter (Semrock FF01-750/SP) as well as a band-pass emission filter (Semrock FF01-510/84), and detected using a multi-alkali photomultiplier (Hamamatsu R3896).

Images were acquired with ScanImage software. The laser intensity was controlled using a combination of an electrically-controlled liquid crystal variable phase retarder (LRC-200-IR1, Meadowlark Optics) and a polarizer beamsplitter (BB-050-IR1, Meadowlark Optics). A 4 μs dwell time was used when imaging. Anatomical images were averages of 2 consecutive images.

#### Confocal system

A confocal Microscope Zeiss LSM 510 was used to examine fixed slices. YFP was excited with the 488 nm beam from an Argon/2 laser though a dichroic HFT488 and a Zeiss Plan Neofluar 20x/0.50 Objective. Epi-fluorescence was filtered through the dichroic HFT488 and a long pass filter LP505.

### Photo-stimulation

#### Two-photon holographic stimulation with amplified laser system

**Figure 1:**
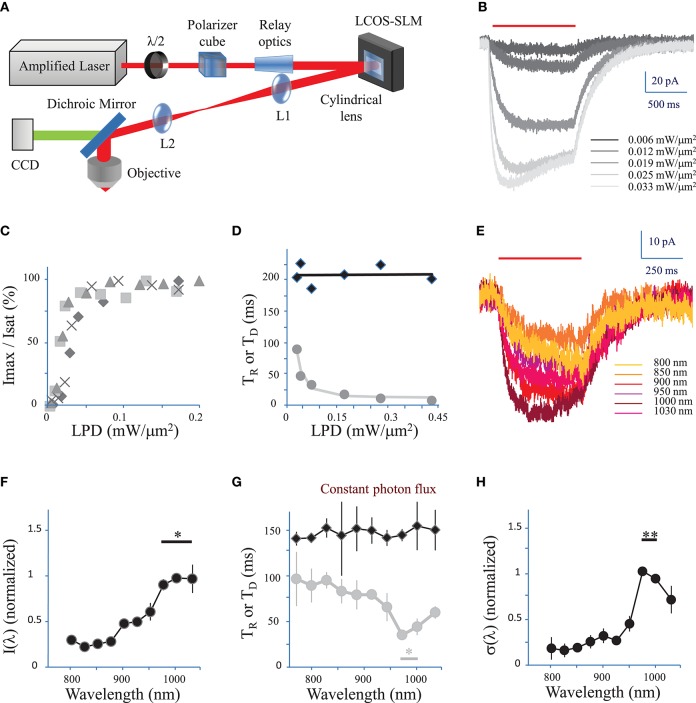
**Characterization of currents evoked by 2P holographic stimulation and two-photon excitation spectrum**. **(A)** Simplified scheme of the 2P holographic stimulation microscope. The output beam from an Ytterbium-doped photonic crystal fiber amplifier Laser System was attenuated by rotating a half-waveplate combined to a polarizer cube. The beam was relayed and expanded to cover the surface of an LCoS-SLM (Hamamatsu X10468-07). Zero-order excitation was suppressed by aberrating it with a cylindrical lens. Then the SLM was imaged through a telescope (L1, L2 lenses) at the back focal plane of an IR antireflection coated water-immersion objective (Nikon CFI APO 40X WI NIR NA 0.80) mounted on an upright microscope (Scientifica). **(B)** CHO cells were voltage clamped at −40 mV and the current response to 2P holographic stimulation (1000 ms, red bar, λ = 1030 nm) with patterns matching the cell shape was recorded. Evoked response for increasing Laser Power Densities (LPDs) in a single cell. Average of *n* = 3 repetitions. **(C)** The maximum current (*I*_*max*_) reached by 2P holographic stimulation at 1030 nm of 4 CHO cells was normalized to the saturation current (*I*_*sat*_) and plotted vs. LPD. Average of *n* = 3 repetitions for each cell and LPD. Data recorded above a LPD of 0.2 mW/μm^2^ was not shown as all cells had reached the saturation current. **(D)** Rise time (*T*_*R*_, gray dots) and decay time (*T*_*D*_, black diamonds) for 2P holographic stimulation of a single CHO cell. Average of *n* = 3 repetitions. *T*_*D*_ and *T*_*R*_ were fit, respectively, by a linear function (black line) and following Equation 4 (in Methods see Section Currents evoked by photo-stimulation of opsins) (gray line). **(E)** CHO cells were voltage clamped at −40 mV and the current response to 2P Generalized Phase Contrast (GPC) stimulation (400 ms, red bar) was recorded at a wavelength range from 720 to 1030 nm, while keeping the photon flux constant (2.7 × 10^26^ photons/s/m^2^). Average of *n* = 3 repetitions for each wavelength. **(F)** The maximum current reached by 2P GPC stimulation was normalized to the current generated with stimulation at 1000 nm and plotted vs. excitation wavelengths. A constant photon flux of 2.7 × 10^26^ photons/s/m^2^ was used. The current generated between 975 and 1030 nm (^*^) was significantly larger than currents generated at other wavelengths (*p* = 0.05, Wilcoxon paired *T*-test; *n* = 6 cells). **(G)** Decay time (*T*_*D*_, black diamonds) and rise time (*T*_*R*_, gray dots) for a constant photon flux of 2.7 × 10^26^ photons/s/m^2^ for 2P holographic stimulation of CHO cells. Average of *n* = 6 cells. *T*_*R*_ between 975 nm and 1000 nm (^*^) was significantly larger than currents generated at other wavelengths (*p* = 0.05, Wilcoxon paired *T*-test; *n* = 6 cells). **(H)** The 2P cross-section, normalized to its value for excitation at 1000 nm, was plotted vs. excitation wavelengths. The 2P cross-section at 975 and 1000 nm (^**^) was very significantly larger than the cross-section at other wavelengths (*p* = 0.01, Wilcoxon paired *T*-test; *n* = 11 cells).

The 2P computer-generated holographic (CGH) system (Figure 1A) has been already described in (Ronzitti et al., 2016). Briefly, the system is based on an LCoS-SLM (Hamamatsu X10468-07) illuminated by an Ytterbium-doped photonic crystal fiber amplifier laser system (Satsuma HP, Amplitude Systems; pulse width 250 fs, tunable repetition rate 500–2000 kHz, gated from single shot to 2000 kHz with an external modulator, maximum pulse energy 20 μJ, maximum average power 10 W, λ = 1030 nm) operated at 500 kHz. The output of the amplifier was relayed and expanded to cover the SLM surface. Zero-order excitation was suppressed by a cylindrical lens (Hernandez et al., 2014). Then the SLM was imaged through an afocal telescope (*f*_1_ = 500 mm and *f*_2_ = 300 mm) at the back focal plane of an IR antireflection coated water-immersion objective (Nikon CFI APO 40X WI NIR NA 0.80) mounted on an upright microscope (Scientifica). Total power at the exit of the objective was 2 W.

We used an amplified pulsed laser (500 kHz, 20 μJ) instead of a conventional femtosecond oscillator, to maximize the peak power and minimize the average power necessary to photo-stimulate a single cell. This solution has previously been implemented to maximize the efficiency of excitation in conventional 2P microscopy imaging (Theer and Denk, 2006).

The SLM was controlled by a custom-designed software (Lutz et al., 2008) based on a Gerchberg and Saxton iterative algorithm, which converts an arbitrary intensity pattern on the sample plane to a specific phase profile to be addressed at the SLM plane. Photo-stimulation light power densities (LPDs) given in the text correspond to values after the objective, at the surface of samples.

#### Two-photon holographic stimulation with standard femtosecond oscillator

To assess the impact of using an amplified pulsed laser we also tested AP generation using ReaChR 2P holographic stimulation with a standard femtosecond oscillator. To do that we used a system similar to the previous one but with a 80 MHz Ti:Sapphire Laser (Mai Tai Deepsee, Newport-Spectra Physics) delivering 100 fs impulsions as a light source.

#### Two-photon GPC stimulation for measurement of spectra

Spectra were obtained via a Generalized Phase Contrast (GPC) shaped illumination (Gluckstad, 1996) through a tunable wavelength Ti:Sapphire laser. GPC, with respect to CGH, provides homogeneous intensity patterns and therefore the same excitation intensity to light-gated channels distributed all over the membrane (Papagiakoumou et al., 2010; Bañas et al., 2014). The optical system is similar to the one described in (Papagiakoumou et al., 2010) with the difference that the grating for temporal focusing was replaced by a mirror. Briefly, the expanded (4x) beam of a Ti:Sapphire Laser (Mai Tai Deepsee, Newport-Spectra Physics) was phase modulated by a Liquid Crystal on Silicon Spatial Light Modulator (LCOS-SLM) (Hamamatsu Photonics X10468-02) placed at the entrance of a GPC common path interferometer and controlled by a custom-designed software. The interferometer is based on a 4*f* (*f* : focal length) imaging path (*f*_1_ = 300 mm, *f*_2_ = 400 mm) with a half-wave phase shifting contrast filter (PCF) at the confocal plane between the lenses. The output pattern of the interferometer is demagnified at the sample plane by a telescope made of a *f* = 500 mm lens and an Olympus LUMPLF 40x W/IR, NA 0.8 objective. The PCF is a patterned glass-based phase mask containing circular pits (the size of the pit used corresponds to 70 μm in diameter). It is designed to provide a π-phase shift for 900 nm wavelength. The robustness of GPC light patterning under a broad wavelength range (Palima and Glückstad, 2008), allowed us the spectra acquisition. The femtosecond laser was tuned from 800 to 1030 nm. During experiments, the wavelength was varied non-monotonically in order to avoid any bias in the spectra.

### Electrophysiology

Patch pipettes were pulled from borosilicate glass capillaries (outer diam. 1.5 mm, inner diam. 0.86 mm Harvard apparatus). Voltage and current signals were recorded using a MultiClamp 700B amplifier (Molecular Devices)), in the whole-cell recording configuration, low-pass filtered at 10 kHz, digitized at 20–40 kHz and recorded using Neuromatic (http://www.neuromatic.thinkrandom.com, written in Igor Pro). Data was analyzed using Neuromatic or pClamp (Axon Instruments) and Microsoft Excel. Membrane potentials are given without correction for the liquid junction potential.

#### Cultured cells

Cultured cells were transferred for recording in a chamber mounted on the headstage of an upright microscope (Scientifica or Olympus BX50WI for spectra measurements). Recordings were performed at room temperature (18–22°C). The extracellular medium during electrical recording was of the following composition (in millimoles of compound used to make a liter of solution): 140 NaCl, 5 KCl, 2 CaCl_2_, 1 MgCl_2_, 20 HEPES, 25 Glucose. pH adjusted to 7.5. Patch pipettes, filled with an intracellular solution was composed of (in millimoles of compound used to make a liter of solution): 140 KCl, 2 MgCl_2_, 2 Mg ATP, 0.4 Na GTP, 10 HEPES, 20 BAPTA. PH adjusted to 7.3, and had a resistance that ranged from 4.5 to 10 MΩ. Experiments were performed in the dark to avoid any direct stimulation of cells by ambient light.

#### Brain slices

Brain slices were transferred for recording in a chamber mounted on the headstage of an upright microscope (Scientifica). Cortical layer 2/3 was localized using infrared illumination and custom-made contrast. Slices were then washed for 15 min with external solution saturated with 95% O_2_/5% CO_2_ containing (in millimoles of compound used to make a liter of solution): 125 NaCl, 2.5 KCl, 26 NaHCO_3_, 1.25 Na_2_H_2_PO_4_, 25 Glucose, 0.5 Ascorbic Acid, 1.5 CaCl_2_ and 1 MgCl_2_. For recordings, slices were continuously perfused with the same external solution at room temperature (18–22°C). Patch pipettes, filled with an intracellular solution of the following composition (in millimoles of compound used to make a liter of solution): 130 K-Gluconate, 7 KCl, 10 HEPES, 4 Mg ATP, 0.3 Na GTP, 10 Na phosphocreatine, pH adjusted to 7.35, and had a resistance that ranged from 5 to 10 MΩ. The slices were used for 1–5 h. Recorded cells were stable for at least 1 h. Experiments were performed in the dark to avoid any direct stimulation of cells by ambient light.

Cells were characterized based on their location and classified based on their firing pattern in response to steps of somatic current injection (Beierlein et al., 2003; Xu et al., 2006). We assessed the presence / absence of a fast and a slow after-hyperpolarization (AHP) based on the AHP rise time. Cells were considered as having a fast AHP when their AHP had a component with a rise time shorter than 10 ms and a slow AHP when their AHP had a component with a rise time larger than 10 ms. Cells were classified as pyramidal cells when they did not have fast AHP but had a slow AHP. Cells were classified as fast-spiking interneurons when they had a fast AHP and when they did not have a slow AHP. Their fast AHP amplitude was 18 ± 4 mV (*n* = 12). Cells were classified as low threshold spiking interneurons when they had a fast and a slow AHP. Their fast AHP amplitude was 10 ± 5 mV (*n* = 4). Many LTS cells evoked rebound spikes in response to hyperpolarizing current.

#### Currents evoked by photo-stimulation of opsins

Following the models previously suggested for ChR2 (Rickgauer and Tank, 2009) for short illumination time, temporal photocurrent traces can be modeled by assuming a 2-state open (O) and closed (C), model: C ↔ O. With such a model the time-dependence of evoked currents will be determined by single exponentials.

Based on these assumptions, the change in the open channel density *[O]* at time *t*, is given by the difference between the rate at which channels open and the rate at which they close. The rate at which channels open can be expressed as the product of the rate at which ReaChR absorbs excitation photons: σ_*m*_(λ) *LPD*^2^ *[C]* (Xu and Webb, 1996), where LPD is the Laser Power Density, *[C]* is the closed channel density at time *t*, σ_*m*_(λ) is the wavelength dependent excitation cross-section of ReaChR, by α(λ) the wavelength dependent quantum yield. The product ασ_*m*_(λ) is called the “action cross-section”, σ(λ) and depends on the excitation wavelength, λ used for illumination (Zipfel et al., 2003). The rate at which the channels close is given by the product of the off-rate coefficient *k*_*off*_ by *[O]* (Rickgauer and Tank, 2009).

Therefore:

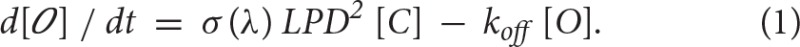



As [*C*] + [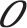] = c, with c = total density of opsins:

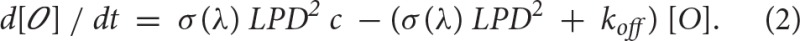



Solving Equation (2) gives for any time *t* before the end of the excitation pulse:

(3) $$[O]\;=\;O_{max}\;(1\;-e^{-t/T_{R}}),$$

where

(4) $$T_{R}\;=\;1\;/\;(\sigma (\lambda )\;LPD^{2}\;+\;k_{off})$$

is the expression for the rise time and

(5) $$O_{max}\;=c\sigma (\lambda )\;LPD^{2}\;/\;(\sigma (\lambda )\;LPD^{2}\;+\;k_{off})$$

describes the maximum number of channels opened at saturation.

As the current induced by photo-stimulation is the product of the single channel conductance and the density of opened channels,

(6) $$I\;=\;I_{max}(\lambda )(1-e^{-t/TR})$$

with

(7a) $$I_{max}(\lambda )\;=\;I_{sat}\;\sigma (\lambda )\;LPD^{2}\;/\;(\sigma (\lambda )\;LPD^{2}\;+\;k_{off})$$

and *I*_*sat*_ is the saturation current.

For *LPD* < < (*k*_*off*_ / σ(λ))^1/2^, i.e., for LPDs inducing currents far below *I*_*sat*_,

(7b) $$I_{max}(\lambda )\;\approx \;I_{sat}\;\sigma (\lambda )\;LPD^{2}\;/\;k_{off}$$

and the plot of *I*_*max*_(λ) as a function of λ enables to obtain the corresponding dependence of the excitation cross-section, σ(λ). Normalizing to the peak (reached at λ = 1000 nm) we have:

(8) $$\sigma (\lambda )\;/\;\sigma (1000)\;\approx \;I_{max}(\lambda )/I_{max}(1000).$$

Alternatively, by setting

$$T_{D}\;=\;1\;/\;k_{off},$$

where *T*_*D*_ is the decay time constant, Equation (4) becomes:

(9) $$1/T_{R}\;-\;1/T_{D}\;=\;\sigma (\lambda )\;LPD^{2}$$

and assuming α independent of λ, we can derive a dependence of the excitation cross-section on the excitation wavelength valid also for high LPD values:

(10) $$\sigma (\lambda )\;/\;\sigma (1000)={\left(1/T_{R}-1/T_{D}\right)}_{\lambda }\;/\;{\left(1/T_{R}-1/T_{D}\right)}_{1000}.$$

Furthermore, the LPD is linked to the photon flux Φ by the equation:

(11) $$LPD\;=\;hc_{0}\;\Phi \;/\lambda$$

where *h* is the Planck constant and c_0_ the speed of light.

Using a constant photon flux Equations (8) and (10) become respectively:

(12) $$\sigma (\lambda )\;/\sigma (1000)\;\approx \;{\left(\lambda /1000\right)}^{2}I_{max}(\lambda )\;/\;I_{max}(1000),$$

and

(13) $$\begin{array}{l} \sigma (\lambda )\;/\;\sigma (1000)={\left(\lambda /1000\right)}^{2}\;{\left(1/T_{R}\;-\;1/T_{D}\right)}_{\lambda }\;/\\ \;{\left(1/T_{R}\;-\;1/T_{D}\right)}_{1000}. \end{array}$$

#### Voltage clamp measurements of currents evoked by photo-stimulation of opsins

CHO cells were voltage-clamped at −40 mV. Cortical cells were voltage-clamped at −70 mV. We adjusted the length of the photo-activation pulse (300 to 1000 ms) depending on the LPD such as the maximum current was reached but inactivation is minimized. Each trial was repeated 3 times and the average of the 3 repetitions was used for further analysis.

The photo-stimulation evoked currents were filtered 100 times with a binomial filter and *I*_*max*_*(*λ*)* was determined as their maximal absolute value. The rise time constant *T*_*R*_ and the decay time constant *T*_*D*_ were determined by fitting the rising phase and the decaying phase of the trace with single exponentials and extracting the 1/e time constants, *T*_*R*_ and *T*_*D*_.

*I*_*sat*_ was determined by fitting *I*_*max*_*(*λ*)* vs. LPD according to (Equation 7). The distribution of *I*_*sat*_ over the population of cells tested was calculated, as well as its cumulative distribution. The LPD at current saturation was defined as the LPD used to reach a current equal to 95% of *I*_*sat*_.

#### Voltage response to photo-stimulation of opsins

Each trial was repeated 3 times. APs were detected for each trial and their average frequency calculated. Conditions for which at least 2 trials out of the 3 induced APs or trains of APs were considered as positive. The average frequency of the 3 repetitions was used for further analysis.

Data through the manuscript is expressed as mean ± s.d.

## Results

### Two-photon current generation and two-photon action spectra in cell culture

We first tested 2P stimulation of ReaChR in cell culture under 2P holographic illumination provided by an amplified laser source (Figure 1A). Patterns of increasing LPDs and matching the cell shape (65 ± 13 μm^2^, *n* = 4 cells) evoked currents of increasing amplitude (Figure 1B) that saturated [Figure 1C, in Methods see Section Voltage clamp measurements of currents evoked by photo-stimulation of opsins and Equation (7)] at 113 ± 54 pA (*n* = 4 cells) for a LPD of 0.10 ± 0.03 mW/μm^2^ (*n* = 4 cells) after the objective. These results demonstrate that ReaChR is sensitive to 2P stimulation even at low LPDs.

The Rise time (*T*_*R*_) (defined in Methods see Section Voltage clamp measurements of currents evoked by photo-stimulation of opsins) varied from 8 ± 2 ms for a LPD of 0.3 ± 0.1 mW/μm^2^, to 145 ± 39 ms for a LPD of 0.008 ± 0.002 mW/μm^2^ (*n* = 4 cells) (Figure 1D). Decay time (*T*_*D*_) (defined in Methods see Section Voltage response to photo-stimulation of opsins) was independent on LPD and equal to 212 ± 24 ms (*n* = 4 cells) (Figure 1D). These values are consistent with the slow kinetics of ReaChR found under 1P illumination (at current saturation: *T*_*R*_ = 20 ± 0.6 ms; *T*_*D*_ = 137 ± 7.1 ms Lin et al., 2013).

We then investigated the dependence of 2P-photocurrent on the excitation wavelength for a range of wavelengths from 720 to 1030 nm (Figure 1E). This was achieved by illuminating the cells with a circular shape generated using the GPC method coupled to a tunable Ti-Sapphire laser (see Section Methods) and a constant photon flux of 2.7 × 10^26^ photons/s/m^2^ after the objective, corresponding to an LPD of 0.04 mW/μm^2^ at 1030 nm. The maximum photocurrent was reached at wavelengths between 975 and 1030 nm (*n* = 6 cells) (Figure 1F; *p* = 0.05, Wilcoxon paired *T*-test). The Rise time (*T*_*R*_) reached a minimum of 41 ± 9 ms between 975 and 1000 nm (*n* = 6 cells) (Figure 1G, *p* = 0.05, Wilcoxon paired *T*-test). Decay time (*T*_*D*_) was independent on wavelength (Figure 1G).

In order to keep a good signal to noise ratio for photocurrents registered at the tail of the action spectra (λ < 850 nm), we also used relative high photon flux (7.5 × 10^26^ photons m^−2^ s^−1^ after the objective) which was close to the saturation power for excitation wavelength within the absorption peak. To derive the exact wavelength dependence of the 2P-cross-section, σ(λ), we calculated its expression as a function of the rise and decay times (see Section Methods, Equations 9, 10). The resulting plot revealed a peak at 975–1000 nm (*n* = 11 cells) (Figure 1H; *p* = 0.01, Wilcoxon paired *T*-test).

These results demonstrate that at the wavelength of the employed amplified laser (1030 nm), currents close to 70% of the maximum achievable current (λ = 975 nm) can be evoked.

### Two-photon holographic activation of ReaChr-expressing neurons in brain slices

**Figure 2:**
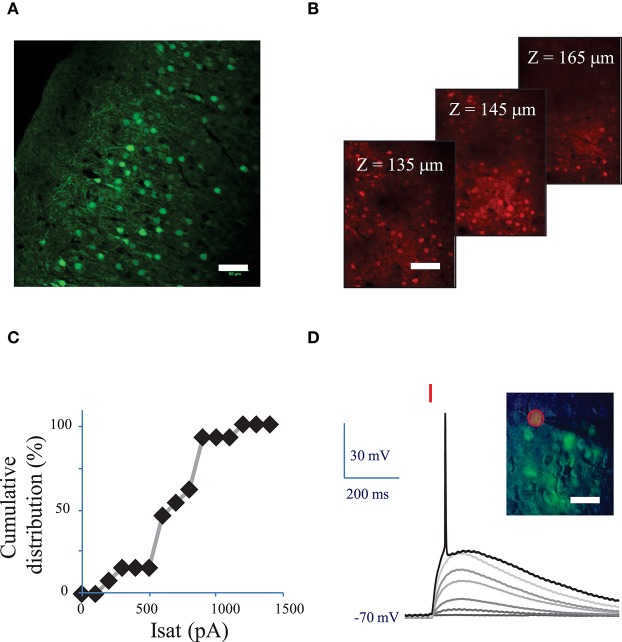
**Conditions to evoke an action potential in neurons of mouse visual cortex Layer 2/3 with a fiber amplified Laser System at 1030 nm**. **(A)** Maximum intensity projection of a z-stack of confocal images of a fixed sagittal brain slice from mouse injected with AAV1 CamKII-ReaChR-p2A-YFP, 2 weeks after injection. Scale bar: 50 μm. **(B)** 2P microscopy images of visual cortex layer 2/3 from an anesthetized mouse (1% isoflurane) injected with AAV1 Ef1α-ReaChR-p2A-dTomato, 7 weeks after injection. Scale bar: 50 μm. Selected images from a stack of 101 images (100 to 200 μm under the surface of the brain). **(C)** Cumulative distribution of the saturation current (in Methods see Section Voltage clamp measurements of currents evoked by photo-stimulation of opsins) generated by 2P holographic stimulation in L2/3 cells when cells were voltage-clamped at −70 mV. **(D)** Voltage response of a L2/3 pyramidal cell to a series of 10-ms long 2P holographic spots (15 μm diameter, over the cell soma) of constant Laser Power Density (LPD) (red bar). Steady state current injection was used to keep the cell voltage at −70 mV in resting conditions. The LPD was increased from 0 to 0.014 mW/μm^2^ within the series until an action potential (AP) was evoked. Inset: overlay of infrared (IR) slice image (grayscale), fluorescence (green) and 2P stimulation mask (red circled area) of V1 layer 2/3 in an acute sagittal brain slice from mouse injected with AAV1 CamKII-ReaChR-p2A-YFP, 5 weeks after injection. Scale bar: 30 μm.

ReaChR was expressed in the visual cortex of mice using AAV1 CamKII-ReaChR-p2A-YFP or AAV1 Ef1α-ReaChR-p2A-dTomato (see Section Methods). Constructs with the p2A sequence between ReaChR and the fluorescent reporter facilitate the identification of opsin-expressing cells (Castelló et al., 2011; Prakash et al., 2012) as they allow for the reporter to be freely-diffusing in the intracellular medium, while the opsin is targeted to the membrane. Therefore, opsin-expressing cells are filled with the fluorescent reporter and can be easily identified under 1P or 2P imaging. Five or six weeks after injection of AAV1 CamKII-ReaChR-p2A-YFP and 6–8 weeks after injection of AAV1 Ef1-ReaChR-p2A-dTomato, cortical layer 2/3 was relatively sparsely labeled (Figures 2A,B). In agreement with previous findings (Lin et al., 2013), cells expressing ReaChR had normal morphology and physiological membrane properties, suggesting minimal toxic effects of expression. Indeed their resting potential was −70 ± 7 mV (*n* = 35) for pyramidal cells, −64 ± 7 mV (*n* = 11) for fast spiking (FS) cells and −66 ± 10 mV (*n* = 4) for low threshold spiking (LTS) cells.

Fifteen micrometer diameter 2P holographic spots were targeted at the somas of L2/3 cortical cells expressing ReaChR in acute brain slices for 0.1–1 s. Evoked currents saturated on average at 740 ± 210 pA (*n* = 12 pyramidal cells) (Figure 2C) for a LPD of 0.04 ± 0.02 mW/μm^2^) (see Section Methods). *T*_*R*_ varied from 28 ± 15 ms for a LPD of 0.06 mW/μm^2^ (*n* = 5 cells), to 55 ± 29 ms for a LPD of 0.02 mW/μm^2^ (*n* = 10 cells).

These results demonstrated that 2P illumination of ReaChR enables generation of currents in the nanoampere scale, in neurons, at LPDs below 0.1 mW/μm^2^ using 1 to 10 ms long 2P stimulation pulses.

### Characterization of light power density necessary to reach the AP threshold using amplified laser or Ti:Sapphire oscillator

Next we investigated the 2P stimulation conditions to evoke an AP in cortical layer 2/3 cells following infection with either of the ReaChR-carrying viruses. 15-μm diameter 2P-holographic spots were applied for 10 ms to depolarize the cells and the laser power was increased until an AP was evoked (Figure 2D). The AP threshold was reached for LPDs of 0.03 ± 0.02 mW/μm^2^ (*n* = 26) in pyramidal cells, 0.02 ± 0.02 mW/μm^2^ (*n* = 8) in FS cells and 0.02 ± 0.02 mW/μm^2^ (*n* = 7) in LTS cells. This is about half the LPD needed to reach current saturation.

**Figure 3:**
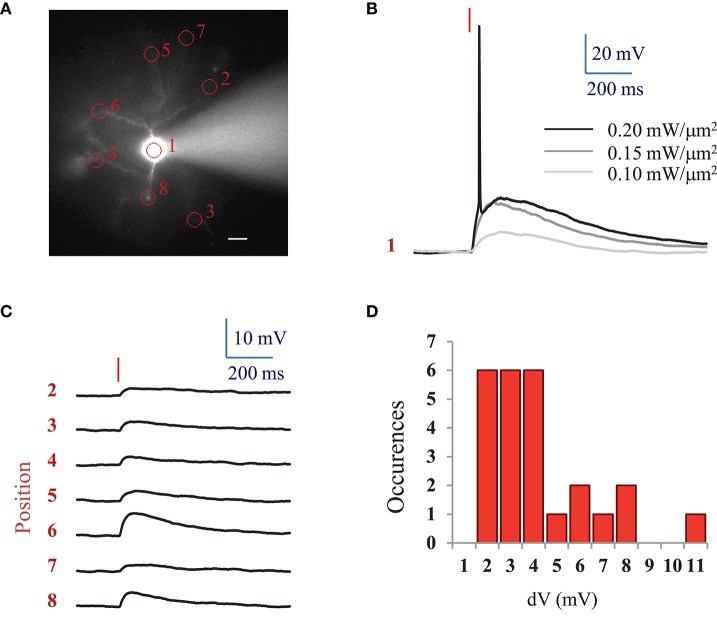
**AP threshold with standard femtosecond Ti:Sapphire oscillator and spatial specificity of 2P holographic stimulation of ReaChR**. **(A)** Widefield fluorescence image of L2/3 ReaChR-expressing neuron filled with 20 μM Alexa 594. Scale bar: 15 μm. **(B)** Voltage response (single recording) to a 10 ms long (red line) 2P holographic stimulation with a standard femtosecond Ti:Sapphire oscillator at 950 nm using a circular spot on the cell soma (1 in panel A, 15 μm diameter) for increasing LPDs. **(C)** Average voltage response (*n* = 3 repetitions) to a 10 ms long (red line) 2P holographic stimulation with a standard femtosecond Ti:Sapphire oscillator at 950 nm on numbered circular spots (15 μm diameter) in panel A. LPD: 0.2 mW/μm^2^. **(D)** Distribution of average voltage response (*n* = 3 repetitions, 25 spots, 3 cells) to a 10 ms long (red line) 2P holographic stimulation with a standard femtosecond Ti:Sapphire oscillator at 950 nm on cell processes. Distance from the soma from 15 to 100 μm, as shown in panel A. LPD corresponding to the one used to reach the AP threshold for 2P stimulation on the cell soma for each cell (0.15 to 0.25 mW/μm^2^).

For comparison, we also investigated ReaChR excitability using a standard femtosecond Ti:Sapphire oscillator (MaiTai-DeepSee, Spectra-Physics, in Methods see Section Two-photon holographic stimulation with standard femtosecond oscillator). We found that an average LPD of 0.2 ± 0.04 mW/μm^2^ (*n* = 5 cells) was necessary to reach the AP threshold with at 950 nm and an excitation spot of 15 μm in diameter for 10 ms illumination pulses (Figures 3A,B).

Interestingly, at the LPD used at AP threshold, only 2P stimulation on the cell body resulted in an action potential. However, 2P holographic stimulation of the processes using the same spot resulted in a 3.72 ± 2.29 mV depolarization (*n* = 3 cells, average of 3 to 8 spots at distances of 15 to 100 μm from the soma) (Figures 3A,C,D).

These results demonstrated that the combination of an amplified laser with ReaChR enables reproducible (*n* ≥ 3) AP generation in 97% of ReaChR expressing-cells in which it was tested, using an average LPD of 0.025 ± 0.022 mW/μm^2^ for an average depth of 48 ± 19 μm (*n* = 43 cells). This corresponds to less than 6 mW of total power per cell.

### Conditions to minimize action potential latency and temporal jitter

**Figure 4:**
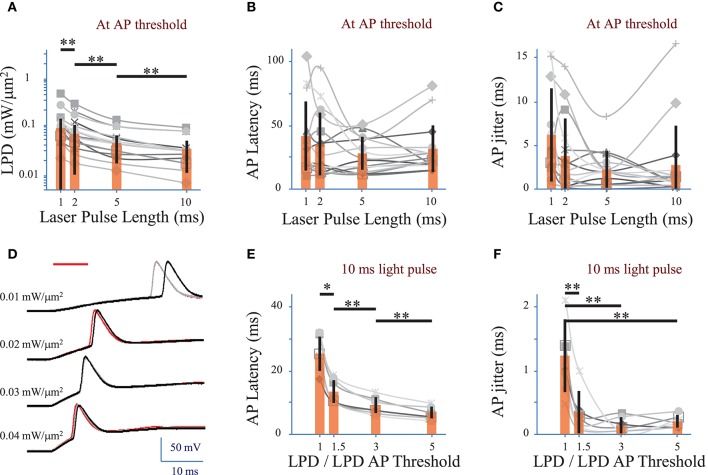
**Optimization of parameters to evoke an action potential in neurons of mouse visual cortex Layer 2/3 with a fiber amplified Laser System at 1030 nm**. **(A)** Laser power density (LPD) necessary to evoke an AP with 1 to 10 ms long 2P holographic spots (15 μm diameter, over the cell soma) (*n* = 16 cells, average of 3 repetitions for each). Individual cells (gray), average (orange bars) and s.d. (error bars). (^**^) (*p* = 0.01, Wilcoxon paired *T*-test). **(B)** Latency between the AP evoked by 1 to 10 ms long 2P holographic spots (15 μm diameter, over the cell soma) at the LPD for AP threshold and the onset of the 2P stimulation (*n* = 16 cells, average of 3 repetitions for each). Individual cells (gray), average (orange bars) and s.d. (error bars). The latency did not vary significantly with the pulse length at AP threshold. **(C)** Same as **(B)** with AP jitter. The jitter did not vary significantly with the pulse length at AP threshold. **(D)** Voltage response of a L2/3 pyramidal cell infected with AAV1 CamKII-ReaChR-p2A-YFP to a series of 10-ms long 2P holographic spots (15 μm diameter, over the cell soma) of constant Laser Power Density (LPD) (red bar). Steady state current injection was used to keep the cell voltage at −70 mV in resting conditions. Three repetitions for each LPD. **(E)** Latency between the AP evoked by 10 ms long 2P stimulation and the onset of 2P stimulation (*n* = 7 pyramidal cells, average of 3 repetitions for each), at LPDs varying from the LPD necessary to reach the AP threshold to 5 times this value. Example of recording on panel D. Individual cells (gray), average (orange bars) and s.d. (error bars). The latency significantly (^*^) (*p* = 0.05, Wilcoxon paired *T*-test) or very significantly (^**^) (*p* = 0.01, Wilcoxon paired *T*-test) decreased when the LPD was increased. **(F)** Same as **(E)** with AP jitter. The jitter very significantly decreased (^**^) (*p* = 0.01, Wilcoxon paired *T*-test) when LPD was increased in comparison to the jitter at LPD used to reach the AP threshold.

We investigated the effect of the photo-stimulation pulse length (1 to 10 ms) on the spiking performances using cells expressing ReaChR following infection with either of the ReaChR-carrying viruses. At the AP threshold, the LPD was 0.03 ± 0.02 mW/μm^2^ for 5 ms pulses (*n* = 16 cells), 0.06 ± 0.06 mW/μm^2^ for 2 ms pulses (*n* = 16 cells) and 0.09 ± 0.1 mW/μm^2^ for 1 ms pulses (*n* = 14 cells) (Figure 4A and Supplementary Figure 2A for pyramidal cells only), thus very significantly larger than for 10 ms pulses, 0.02 ± 0.02 mW/μm^2^ (***p*** = 0.01, Wilcoxon paired *T*-test; *n* = 16 cells).

APs were generated with a latency, i.e., delay between the photo-stimulation onset and the peak of the AP, of 28 ± 11 ms (*n* = 22 cells), with an AP jitter, i.e., standard deviation of the latency, of 2.4 ± 2.2 ms (for 3 repetitions, average of 22 cells) at AP threshold. At AP threshold, both the latency (*p* > 0.1 Wilcoxon paired *T*-test, *n* = 16 cells) (Figure 4B and Supplementary Figure 2B for pyramidal cells only) and jitter (***p*** > 0.1 Wilcoxon paired *T*-test, *n* = 16 cells) (Figure 4C and Supplementary Figure 2C for pyramidal cells only) were independent of the pulse length.

In pyramidal cells, increasing the LPD above the value used to reach the AP threshold reduced significantly both the AP latency and the AP jitter (Figures 4D–F). The latter could be decreased down to 0.3 ± 0.3 ms (*n* = 7 pyramidal cells, *p* = 0.01, Wilcoxon paired *T*-test) by increasing the LPD by a factor of 1.5. Therefore, reliable and millisecond-range controlled single AP generation could be achieved by using 10-ms-long illumination with 2P holographic spots at LPDs about 1.5 as large as LPDs needed to reach AP threshold, i.e., 0.032 ± 0.019 mW/μm^2^ (*n* = 7 cells), which is still below the saturation LPDs (~0.04 mW/μm^2^). In this dataset, all recorded cells had been infected with AAV1 CamKII-ReaChR-p2A-YFP.

Interestingly ReaChR combined with the efficient current integration under parallel photo-stimulation, enables using photo-stimulation pulses much shorter than the channel rise time (see Table 1), therefore enabling AP generation with millisecond temporal resolution despite the slow on-kinetics of the channels.

**Table 1:** **Properties of ReaChR 2P-holographic activation**.

**Laser Pulse Length (ms)**	**1**	**2**	**5**	**10**
LPD (mW/μm^2^) to reach AP threshold	0.09 ± 0.10 (*n* = 15)	0.061 ± 0.061 (*n* = 17)	0.031 ± 0.024 (*n* = 19)	0.025 ± 0.022 (*n* = 43)
*T_R_* (ms) to reach the maximum current in Voltage Clamp at LPD used to reach AP threshold	30 ± 11 (*n* = 2)	28 ± 15 (*n* = 5)	36 ± 22 (*n* = 8)	55 ± 29 (*n* = 10)

*Mean ± s.d., all cell types*.

### Conditions to evoke a train of action potentials

**Figure 5:**
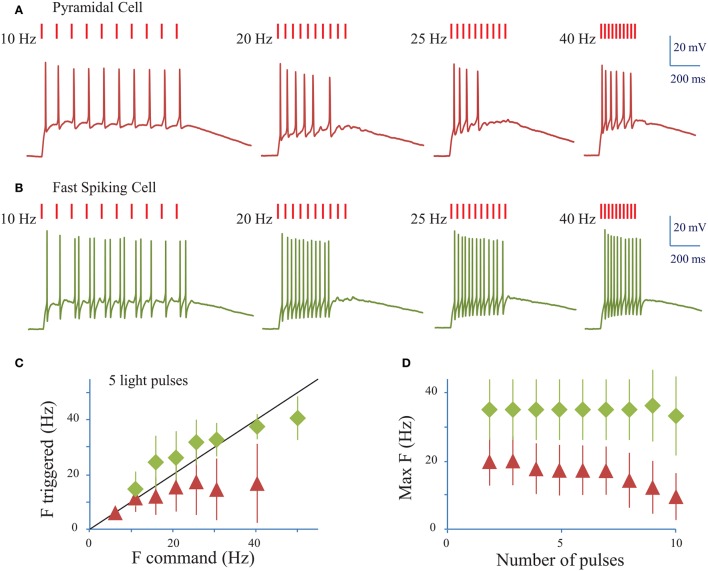
**Conditions to evoke a train of action potentials in neurons of mouse visual cortex Layer 2/3 with a fiber amplified Laser System at 1030 nm**. **(A)** Trains of 2P holographic stimulation pulses were used to evoke trains of APs in single L2/3 cells: example of a pyramidal cell. Trains of 10 pulses at frequencies ranging from 5 to 40 Hz and LPDs at AP threshold were used. **(B)** Same as **(A)** for a fast spiking cell. **(C)** Plot of the maximum AP frequency vs. the photo-stimulation light pulses frequency, for pyramidal cells and LTS cells (red triangles, average of *n* = 19 cells) and FS cells (green diamonds, average of *n* = 4 cells). Black line corresponds to an AP frequency equal to the light pulse frequency. **(D)** Maximum AP frequency reached for trains of a given number of light pulses for pyramidal cells and LTS cells (red triangles, average of *n* = 19 cells) and FS cells (green diamonds, average of *n* = 4 cells).

We next assessed the ability of ReaChR to generate AP trains at various frequencies and on different cell types (Figures 5A,B) following infection with either of the ReaChR-carrying viruses. Trains of up to 10 APs could be generated, but often the cells failed to evoke APs in response to the last few light pulses. On average, 7 ± 3 APs were evoked (*n* = 18 cells, including pyramidal cells and interneurons) by trains of 10 light pulses (10 ms pulse duration) at 20 Hz. The number of APs evoked was strongly dependent on the cell type and, within the same population of cells, on cell membrane properties and ReaChR expression level, which was estimated from the fluorescence of the reporter: at 20 Hz, in FS cells 11 ± 2 APs (*n* = 3 cells) were evoked, in LTS cells 8 ± 3 APs (*n* = 3 cells) were evoked, whereas in pyramidal cells only 6 ± 2 APs (*n* = 11 cells) were evoked. Although synchronicity between light-pulses and spikes can be altered across the overall stimulation train, the frequency of the evoked AP trains remained as large as the frequency of the photo-stimulation train up to 34 ± 12 Hz for FS cells (*n* = 4 cells), up to 22 ± 6 Hz for LTS cells (*n* = 5 cells), and up to 16 ± 9 Hz for RS cells (*n* = 16 cells) for a stimulation train of 5 pulses (Figure 5C) (see Section Methods). This frequency decreased as the number of light pulses increased (Figure 5D). As under 1P illumination, the slow kinetics of the opsin between light pulses generated plateau potentials in all cases and occasionally extra spikes for LTS cells or FS cells (Figure 5B). Therefore, the combination of ReaChR and 2P holographic stimulation allows the reliable generation of 5 AP trains at up to ~15 Hz for pyramidal cells, ~35 Hz for FS cells and ~20 Hz for LTS cells.

## Discussion

We have found that the red-shifted opsin ReaChR can be activated with 2P amplified laser holographic photostimulation when expressed in both cultured cells and mouse neurons in acute brain slices. To our knowledge, this study provides the first demonstration that ReaChR is 2P-sensitive and its activation enables reliable millisecond range control of AP generation with low illumination doses despite its slow activation kinetics.

We have measured the spectrum of ReaChR under 2P excitation. The action cross-section of ReaChR is maximal at 975–1000 nm but it is still 75% of its maximum value at 1030 nm, thus enabling using both a conventional tunable Ti:Sapphire oscillator and a single wavelength amplified laser peaked at 1030 nm. In the first case, LPD necessary to evoke an AP with ReaChR was ~0.2 mW/μm^2^. Because of the higher peak power, the use of an amplified (low repetition rate) laser enabled to reduce the average power necessary to evoke an AP down to 0.03 mW/μm^2^ which is about ten-fold lower than what was required with the conventional laser. This low LPDs corresponds to only 3.5–6 mW per cell. Considering that the total available laser power (after the objective) is ~2 W, these results suggest that, at this depth, it might be possible to achieve simultaneous photo-stimulation of hundreds of cells. For greater depths this number needs to be rescaled to take into account the power losses due to scattering (e.g. at a depth of ~150 μm the effective available power will be reduced by nearly a factor of three; Chaigneau et al., 2011).

Confirming what has been demonstrated using 1P illumination, we found that ReaChR shows slow on- and off-kinetics with 2P stimulation. This could prevent rapid AP generation. However, ReaChR combined with parallel photo-stimulation can surpass the threshold for AP generation with stimulation pulses much shorter than the corresponding rise time. This, similarly to what we demonstrated with C1V1 (Bègue et al., 2013), enabled reducing the effective temporal resolution down to 1 ms. Similar results may be achieved with other slow opsins e.g., CoChR or Chrimson (Klapoetke et al., 2014). We also demonstrated that using pulse durations of 10 ms and light power density ≥1.5-fold of the AP threshold power (~0.02 mW/μm^2^) enabled AP generation with sub-millisecond temporal jitter, as it was achieved with Chronos, an opsin having ten times faster on- and off-kinetics (Ronzitti et al., 2016). This is of the same order of magnitude as the temporal jitter of mono-synaptic events. As 2P holographic stimulation of ReaChR also provides selective AP generation in targeted cells, it provides sufficient spatiotemporal control to trigger AP in chosen presynaptic cells. Thus, it can enable studies concerning postsynaptic activity in well-chosen anatomical configurations. Furthermore, ReaChR and 2P holographic stimulation allows generating trains of 5 APs at up to ~15 Hz for pyramidal cells and LTS cells, and ~35 Hz for FS cells reliably. Therefore, 2P holographic stimulation of ReaChR provides a way to generate repetitive activity in chosen cells and study resulting physiological events.

The measured 2P excitation spectra revealed a peak around 970 nm, and a significantly reduced absorption below 900 nm. These findings indicate a potential efficient use of ReaChR for all-optical monitoring of brain activity if 2P-stimulation of ReaChR is combined with 1P or 2P voltage or calcium imaging (e.g., using OGB bulk loading). Conversely, we expect that the slow off-kinetics and absorption peak at 970 nm will make it difficult to combine ReaChR with GCaMP (Akerboom et al., 2012) or RCaMP (Dana et al., 2016) imaging without evoking spurious photo-stimulation during imaging.

In brain slices, we tested two viral constructs (AAV1 CamKII-ReaChR-p2A-YFP or AAV1 Ef1α-ReaChR-p2A-dTomato) and found in both cases conditions for efficient expression, that didn't alter normal morphological appearances and healthy physiological properties (Supplementary Figure 1) The construct using dTomato as fluorescent reporter could be particularly interesting for in depth cell targeting, or for combination with green calcium indicators. Also it could be used for dual color optogenetics if combined with blue-shifted opsins (e.g., ChR2) conjugated to green fluorescent indicators.

To conclude, 2P holographic stimulation of ReaChR using an amplified laser is very powerful for flexible light patterning using very low average power. These results open the route for high temporal resolution, multiple-cell optogenetic stimulation *in vitro* and *in vivo*.

## Author contributions

EC performed photo-stimulation experiments and data analysis. ER and EP designed and built the microscope for CGH and 2P scanning imaging. EC, ER, and VE designed experiments. MG performed and analyzed photo-stimulation experiments using the mode locked Ti:Sapphire laser. GS and HZ designed and provided constructs and viruses. AB contributed to viral injections and together with ER acquired 2P *in vivo* images. DT and EP built the GPC illumination system and participated in the experiments for the measurements of the absorption spectrum. EC and VE wrote the paper with the contribution of HZ and GS. All authors read and approved the manuscript. VE conceived and supervised the project.

### Conflict of interest statement

The authors declare that the research was conducted in the absence of any commercial or financial relationships that could be construed as a potential conflict of interest.
