# Supplementary material for: Two-Photon Holographic Stimulation of ReaChR
Source: Front Cell Neurosci. 2016 Oct 18;10:234. doi: 10.3389/fncel.2016.00234 (PMC5067533; doi:10.3389/fncel.2016.00234)
Supplement: Supplementary file 1 [file DataSheet1.DOCX]

**Supplementary information**

**Two-photon holographic stimulation of ReaChR**

Emmanuelle Chaigneau^1§^, Emiliano Ronzitti^1^, Marta Gajowa^1^, Gilberto Soler-Llavina^2^, Dimitrii Tanese^1^, Anthony Brureau^1^, Eirini Papagiakoumou^1,3^, Hongkui Zeng^2^ and Valentina Emiliani^1*^

Supplementary Figure 1: Physiological parameters of cells


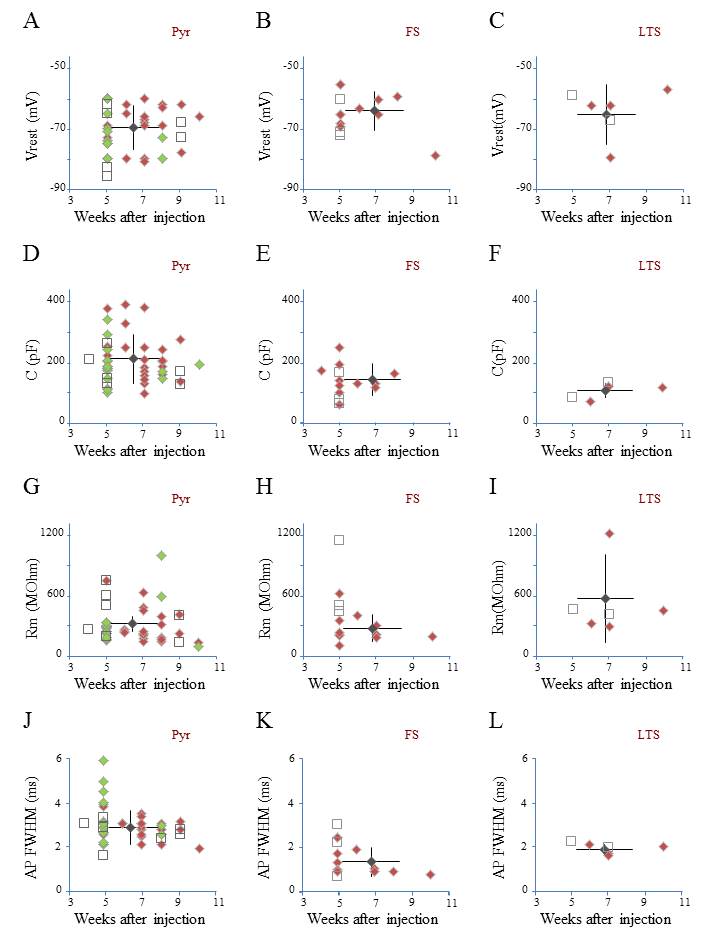


**Legend**:

1. Resting potential in ReaChR expressing pyramidal cells (green diamonds: YFP, n = 14 cells, red diamonds: dTomatoe, n = 21 cells, black diamond: average ± s.d.) and in non-infected cells (black squares, n = 6 cells).
2. Resting potential in ReaChR expressing Fast Spiking (FS) cells (red diamonds: dTomatoe, n = 7 cells, black diamond: average ± s.d.). and in non-infected cells (black squares, n = 3 cells).
3. Resting potential in ReaChR expressing Low Threshold Spiking (LTS) cells (red diamonds: dTomatoe, n = 10 cells, black diamond: average ± s.d.) and in non-infected cells (black squares, n = 2 cells).
4. Cell capacitance of ReaChR expressing pyramidal cells (green diamonds: YFP, n = 16 cells, red diamonds: dTomatoe, n = 25 cells, black diamond: average ± s.d.) and in non-infected cells (black squares, n = 6 cells).
5. Cell capacitance of ReaChR expressing FS cells (red diamonds: dTomatoe, n = 11 cells, black diamond: average ± s.d.) and in non-infected cells (black squares, n = 3 cells).
6. Cell capacitance of ReaChR expressing LTS cells (red diamonds: dTomatoe, n = 4 cells, black diamond: average ± s.d.) and in non-infected cells (black squares, n = 2 cells).
7. Membrane resistance of ReaChR expressing pyramidal cells (green diamonds: YFP, n = 16 cells, red: dTomatoe, n = 25 cells, black diamond: average ± s.d.) and in non-infected cells (black squares, n = 6 cells).
8. Membrane resistance of ReaChR expressing FS cells (red diamonds: dTomatoe, n = 11 cells, black diamond: average ± s.d.) and in non-infected cells (black squares, n = 3 cells).
9. Membrane resistance of ReaChR expressing LTS cells (red diamonds: dTomatoe, n = 4 cells, black diamond: average ± s.d.) and in non-infected cells (black squares, n = 2 cells).
10. Action potential full width half maximum (AP FWHM) of ReaChR expressing pyramidal cells (green diamonds: YFP, n = 15 cells, red: dTomatoe, n = 25 cells, black diamond: average ± s.d.) and in non-infected cells (black squares, n = 7 cells).
11. AP FWHM of ReaChR expressing FS cells (red diamonds: dTomatoe, n = 12 cells, black diamond: average ± s.d.) and in non-infected cells (black squares, n = 3 cells).
12. AP FWHM of ReaChR expressing LTS cells (red diamonds: dTomatoe, n = 4 cells, black diamond: average ± s.d.) and in non-infected cells (black squares, n = 2 cells).

Supplementary Figure 2: Optimization of parameters to evoke an action potential in pyramidal cells of mouse visual cortex Layer 2/3 with a fiber amplified Laser System at 1030 nm


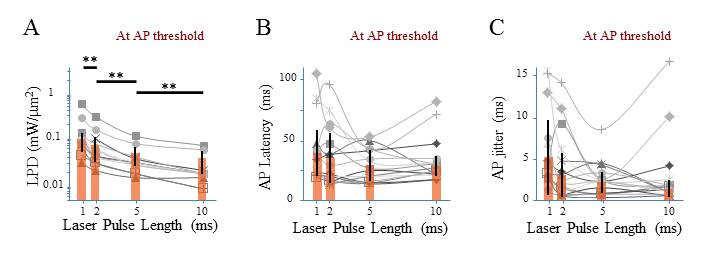


**Legend**:

1. Laser power density (LPD) necessary to evoke an AP with 1 to 10 ms long 2P holographic spots (15 μm diameter, over the cell soma) (n = 13 pyramidal cells, average of 3 repetitions for each). Individual cells (gray), average (orange bars) and s.d. (error bars). (**) (***p*** = 0.01, Wilcoxon paired T-test).
2. Latency between the AP evoked by 1 to 10 ms long 2P holographic spots (15 μm diameter, over the cell soma) at the LPD for AP threshold and the onset of the 2P stimulation (n = 13 pyramidal cells, average of 3 repetitions for each). Individual cells (gray), average (orange bars) and s.d. (error bars). The latency did not vary significantly with the pulse length at AP threshold.
3. Same as (B) with AP jitter. The jitter did not vary significantly with the pulse length at AP threshold.
